# Technological and industrial trends in China’s pharmaceutical sector

**DOI:** 10.3389/fphar.2025.1579037

**Published:** 2025-09-16

**Authors:** Ju Wang, Mengshan He, Fengying Lu, Ying Chen, Hongguang Wang

**Affiliations:** ^1^ The Academy of Chinese Health Risks of West China Hospital, Sichuan University, Chengdu, China; ^2^ The China Strategy Research Center of Peking University, Beijing, China

**Keywords:** China’s pharmaceutical industry, technological trends, industrial trends, industry transformation, global expansion, leading pharmaceutical nation

## Abstract

In recent years, China’s pharmaceutical industry has experienced rapid growth, positioning itself as the world’s second-largest pharmaceutical market and R&D hub. However, the industry faces significant challenges due to policy shifts and the effects of a “capital winter”. This paper provides a comprehensive analysis of the key trends shaping the future of China’s pharmaceutical sector, focusing on the impact of emerging technologies, such as precision medicine and synthetic biology, on drug development processes. It also examines changes in market demand for advanced formulations, chronic disease treatments, and rare disease drugs. Additionally, the paper explores the primary drivers and barriers to the industrial transformation from the perspectives of population aging, industrial restructuring, and internationalization. The findings suggest that technological innovation and industrial upgrading are critical to driving high-quality development in drug research and manufacturing. Achieving this requires coordinated efforts in policy optimization and corporate innovation to overcome technological barriers and promote sustainable, global growth. This study offers theoretical insights and practical recommendations for policymakers and corporate strategists in fostering long-term innovation and competitive advantage in the pharmaceutical industry.

## 1 Introduction

**FIGURE 1:**
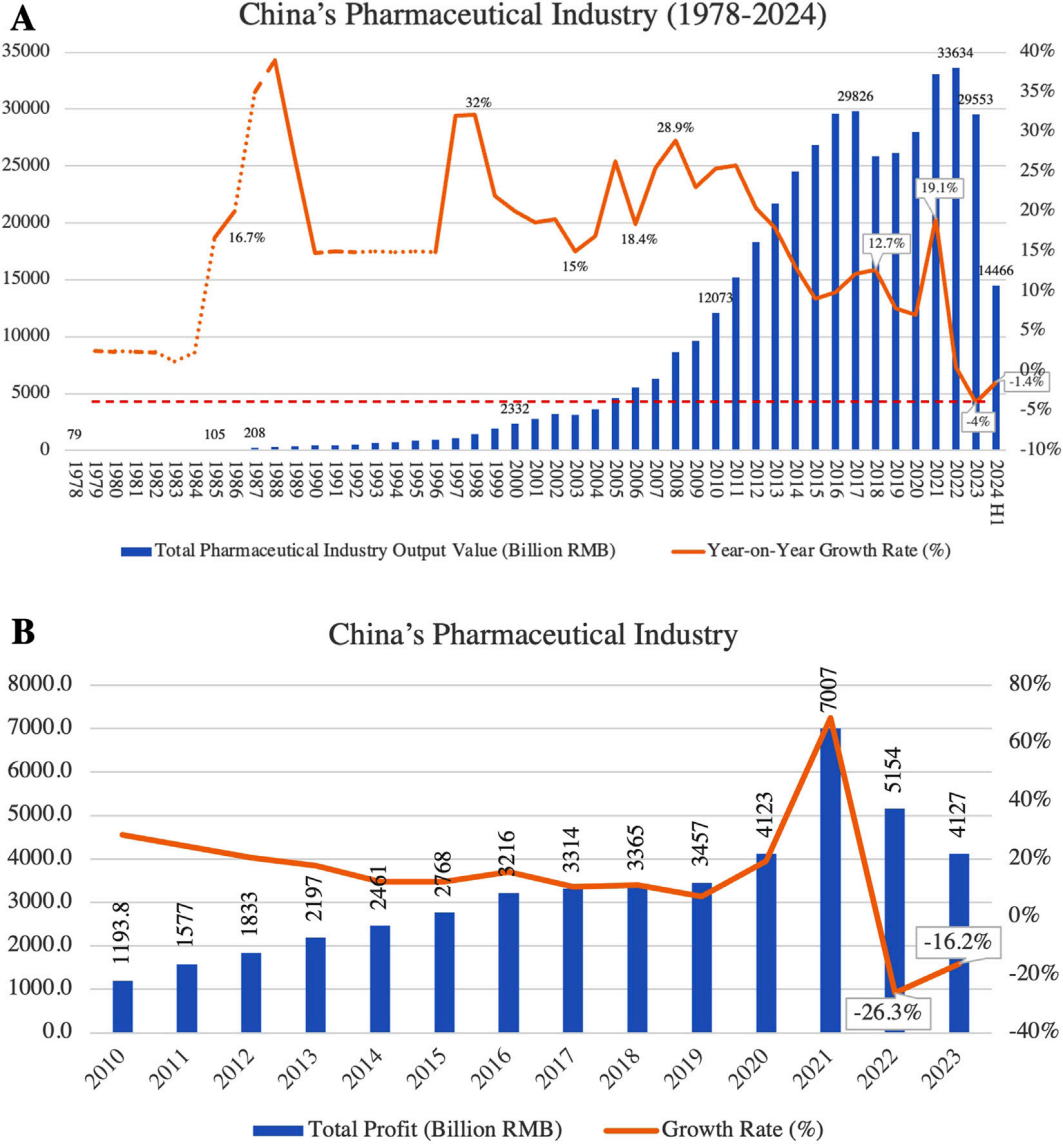
**(A)** Trends in total output value and year-on-year growth rate of China’s pharmaceutical industry (1978–2024); **(B)** total profit and growth rate of China’s pharmaceutical industry (2010–2023). (Source: Annual pharmaceutical yearbook, China pharmaceutical enterprises association).

In recent years, China’s pharmaceutical industry has experienced rapid growth, with the market size expanding from 1.21 trillion RMB in 2010 to 2.97 trillion RMB in 2024. The industry now accounts for 29.5% of the global research and development (R&D) pipeline (ITELINE, 2025), positioning China as the world’s second-largest drug market and a leading hub for drug development and innovation. However, alongside this rapid expansion, the sector faces significant challenges. Policies such as “price negotiations of innovative drugs” and “National Volume-Based Procurement (NVBP)” have led to substantial price reductions, alleviating the financial burden on patients but severely squeezing profit margins for companies. This has particularly affected small and medium-sized generic drug manufacturers (Long et al., 2022; Zheng et al., 2023; Lei et al., 2024). In 2023, the pharmaceutical industry saw negative growth in both revenue and profit, marking the first comprehensive decline in 40 years (Figure 1).

Simultaneously, investor confidence in the pharmaceutical sector has diminished within the capital market, particularly in the innovative drug development segment. Pharmaceutical investment and financing fell back to 2017 levels in 2023, and listed companies faced widespread declines in valuation. The high costs of R&D, lengthy approval cycles and the technological barriers to innovative have pushed many innovative drug companies into financial distress, forcing some startups to scale back or abandon R&D projects and reduce their workforce (Liu and Gao, 2024). This shift from an ‘innovation boom’ to a ‘capital winter’ has placed unprecedented transformational pressure on the industry. In the generic drug sector, the implementation of the “consistency evaluation policy” has heightened competition, driving smaller enterprises out of the market and accelerating industry consolidation (Yang et al., 2024). Overall, China’s pharmaceutical industry is undergoing its most severe downturn in 4 decades.

In navigate these challenges, China’s pharmaceutical industry urgently need to move beyond its traditional reliance on generic drugs and prioritize the development of innovative drugs. Leveraging technological innovation, resource integration, and industrial structure optimization will be key to transitioning toward high-value-added innovative drugs and promoting high-quality development in drug research (Kong et al., 2023). Recently, the Chinese government has introduced a series of policies to facilitate this transformation, including reforms in the drug evaluation and approval system that have significantly shortened the time to market for innovative drugs. The “adjustment of medical insurance catalogue” has further supported the market entry of high-value-added drugs. Moreover, the 14th Five-Year Plan highlights key development areas such as precision medicine, biopharmaceuticals, and advanced pharmaceutical equipment. The government has also implemented multidimensional incentives, including R&D subsidies and priority review channels, aimed at helping innovative pharmaceutical companies overcome bottlenecks (Gao and Chen, 2022). Despite these efforts, the industry still faces challenges, including insufficient policy benefits and limited corporate innovation capabilities. As a result, the shift from generics to innovation remains a central challenge for the pharmaceutical sector’s transformation.

The pharmaceutical industry plays a crucial role in safeguarding public health and social wellbeing. Amid the current downturn, the question of how to guide China’s pharmaceutical industry toward sustainable, high-quality growth has become a critical focus for both academia and industry. This study is both timely and essential, as it addresses a gap in existing literature regarding the dual impact of technological innovation and industrial trends on the industry’s transformation. Particularly how these trends influence its future trajectory and innovation capacity (Chen et al., 2023; Li and Volgina, 2023; Bashirynejad et al., 2024). By analyzing both technological and industrial trends, this study offers valuable insights into the key drivers of transformation within the pharmaceutical industry. It not only provides robust scientific evidence to guide policy formulation and strategic decision-making for business leaders but also serves as a critical reference for academic researchers and industry professionals seeking to understand the dynamics of this rapidly evolving sector.

## 2 Trends in pharmaceutical technology

The pharmaceutical industry is undergoing a pivotal technological transformation, driven by advanced technologies such as precision medicine, synthetic biology, and artificial intelligence (AI) gradually reshaping the drug development landscape. These innovations are driving the realization of personalized treatments and offering novel solutions for complex diseases. This section will examine the latest advancements in these technologies and explore their potential applications within China’s pharmaceutical sector, with a focus on how they influence drug efficacy, pharmacokinetics, and treatment outcomes.

### 2.1 Emerging technologies driving drug development

#### 2.1.1 Personalized treatment and precision medicine

As the incidence of complex diseases such as cancer and cardiovascular diseases continues to rise, the demand for personalized treatment among Chinese patients is steadily increasing. Leveraging advanced technologies like gene sequencing, AI, and big data, personalized treatment is revolutionizing the diagnosis and treatment of complex diseases, including cancer and rare diseases. In 2022, the market size for precision medicine in China surpassed 200 billion RMB, reaching 286 billion RMB in 2024, and is projected to exceed 570 billion RMB by 2030, with an average annual growth rate of approximately 12% (Di Marcantonio et al., 2024). This growth positions precision medicine as one of the main drivers of China’s healthcare sector in the coming years.

Policy support has played a crucial role in accelerating the development of precision medicine. Since the introduction of the “Precision Medicine Strategy” in 2015, the 13th and 14th Five-Year Plans, along with related policies, have significantly advanced the application of technologies such as genetic testing and pharmacogenomics in the personalized treatment of cancer and rare diseases (Di Marcantonio et al., 2024). As of 2023, more than 500 genetic testing products were available in China, with approximately 4,800 related companies operating in the market, demonstrating substantial market potential.

Looking ahead, precision medicine will increasingly focus on technologies such as gene editing, along with targeted drug delivery systems, cell therapy and RNA technologies, to achieve further breakthroughs in cancer immunotherapy and vaccine development. Not only does precision medicine enhance early diagnosis and targeted treatment, but it also drives drug development toward greater efficiency and precision, enabling more targeted pharmacological interventions that improve patient outcomes.

#### 2.1.2 Advancements in synthetic biology and gene editing technologies

Synthetic biology, often dubbed the “third biotechnology revolution”, provides innovative tools for precision drug development through the targeted design of genes, cells, and molecules. The global market for synthetic biology in the pharmaceutical and healthcare industries is expected to experience substantial growth by 2030, with China’s market alone projected to reach $31.5 billion by 2028, growing at an annual rate of 28.65% (SynBioBeta, 2024). Meanwhile, gene editing technologies, such as CRISPR-Cas9, have shown great promise in the development of treatments for genetic diseases like sickle cell disease and β-thalassemia. Notably, products like CASGEVY™ demonstrate the potential of gene editing to propel precision medicine forward (Ag, 2024).

China has placed significant emphasis on synthetic biology and gene editing technologies, making them strategic priorities in the 13th and 14th Five-Year Plans. These technologies enable the creation of novel drug targets and biological pathways, opening up new pharmacological mechanisms for treating previously untreatable diseases. For instance, synthetic biology facilitates the development of biopharmaceuticals that specifically target disease-causing mechanisms at the molecular level, offering more selective and effective treatments. Gene editing enhances these approaches by directly correcting genetic mutations, providing curative therapies for genetic disorders.

#### 2.1.3 AI-enabled drug development

**FIGURE 2:**
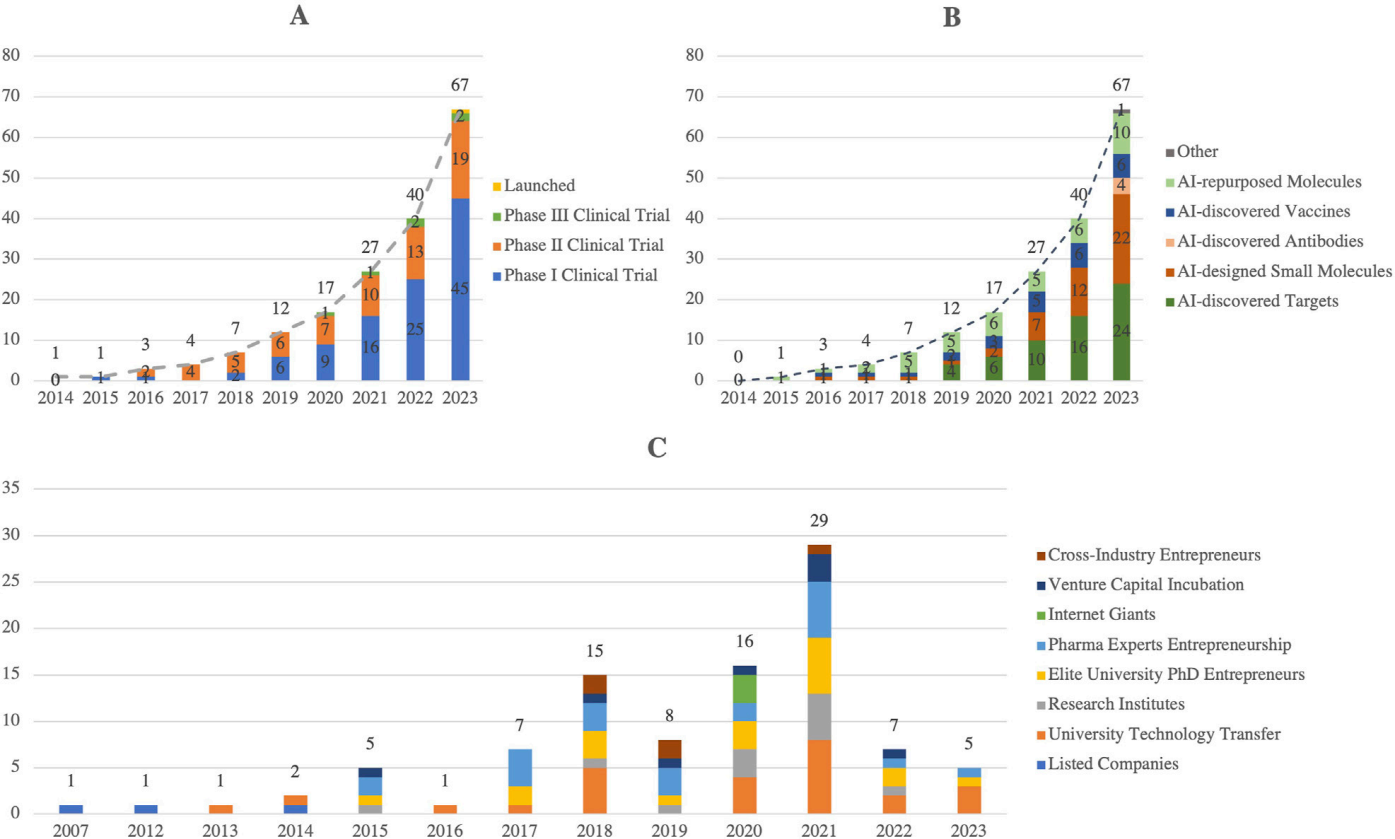
Global AI Drug Development: **(A)** By Clinical Stage, **(B)** By AI Assistance Type, **(C)** Number of AI + Pharmaceutical R&D Companies involved in China (2024). This figure illustrates the rapid progress of AI-assisted drug development globally and its adoption in China. Panel A highlights the steady growth of AI-developed drugs across clinical stages, with a notable increase in launched drugs by 2023. Panel B categorizes the types of AI assistance, including small molecules, antibodies, and drug targets, reflecting diverse applications. Panel C shows the rise of AI + pharmaceutical R&D companies in China, which peaked in 2021 and demonstrates a strong innovation ecosystem. (Source: Drug Discovery Today,2024; Intellectual Medicine Bureau).

Artificial Intelligence (AI) is reshaping drug R&D by improving efficiency, reducing costs, and shortening timelines across multiple stages, including target identification, molecular design, drug screening, and clinical trials (Jiménez-Luna et al., 2021). By 2023, 67 AI-assisted drugs had entered clinical stages globally, covering antibodies, vaccines, and small molecules (Figures 2A,B), with reported success rates of ∼90% in Phase I and ∼40% in Phase II, outperforming traditional approaches (Kp Jayatunga et al., 2024). This success is primarily attributed to AI’s ability to efficiently analyze complex biological data, thereby reducing risks and accelerating early-stage development.

AI’s potential extends beyond enhancing efficiency. It plays a pivotal role in predicting drug-drug interactions, optimizing clinical trial designs, and identifying novel drug targets with high pharmacological potential. For instance, AI-driven drug discovery platforms such as BenevolentAI have led to the development of new treatments for diseases like amyotrophic lateral sclerosis (ALS) and have created possible therapeutic targets, which are currently undergoing tests in clinical trials (Myszczynska, 2021). By incorporating machine learning algorithms into the drug discovery process, AI can predict pharmacodynamics, pharmacokinetics, and the mechanisms of action of drug candidates at an early stage, enabling more precise therapies and efficient drug development. Furthermore, AI’s capacity to integrate and analyze large-scale biological data enables the personalization of drug treatments, making it a key tool in advancing precision medicine (Saini et al., 2025). With its growing capabilities, AI is set to dramatically transform the pharmacological landscape, accelerating the development of safer and more effective drugs.

China’s “AI + Pharma” ecosystem is expanding rapidly, with 98 relevant companies involved by 2024 (Figure 2C) (Sampene and Nyirenda, 2024; Xueqiu, 2024). Notably, Insilico Medicine, a China-based company, advanced an AI-discovered small molecule for idiopathic pulmonary fibrosis (INS018_055) into Phase II clinical trials in 2023 (Xu et al., 2025). Policy support has further promoted the application of AI in drug development, enabling China to advance the creation of efficient and safe drugs, thereby injecting new momentum into the global pharmaceutical market. However, the potential of AI also faces risks related to overhype, particularly concerning data quality, algorithm models, and their alignment with real-world applications. Moving forward, AI is expected to play a pivotal role in reducing R&D costs and improving efficiency through more optimized data integration capabilities, further advancing pharmacological research. Policy support has further catalyzed the application of AI in drug development, enabling China to drive the creation of efficient and safe drugs, thus injecting new momentum into the global pharmaceutical market. However, the full potential of AI faces challenges related to overhype, particularly regarding data quality, algorithm models, and their alignment with real-world applications. Moving forward, AI is expected to play a critical role in reducing R&D costs and enhancing efficiency through optimized data integration capabilities, further advancing pharmacological research.

### 2.2 Drug categories and technological advancements

#### 2.2.1 Cell therapies and RNA technologies accelerating industrialization

Cell therapy, as an advanced therapeutic approach, is progressing from laboratory research to large-scale industrialization. A prominent example of this is CAR-T therapy, which has demonstrated remarkable efficacy in treating hematologic cancers. Research is now expanding to include solid tumors and chronic diseases. From a pharmacological standpoint, CAR-T therapies are revolutionizing cancer treatment by modulating the immune system to specifically target and eliminate cancer cells. The mechanism of action involves immune cell reprogramming to enhance their therapeutic activity, offering personalized treatment options with significantly improved outcomes for patients with certain cancers (Baker et al., 2023).

Meanwhile, RNA technologies are advancing vaccines and antiviral drug development. Nucleic acid vaccines played a pivotal role in the global response to the COVID-19 pandemic. These vaccines utilize RNA molecules to stimulate immune responses without introducing live pathogens, resulting in safer and more efficient prophylactic therapies (Walsh et al., 2020). Moving ahead, RNA technology holds significant potential for applications in cancer vaccines and treatments for rare diseases. The precise delivery and activation of RNA payloads will enable targeted drug delivery, enhancing both the efficacy and safety profile of treatments.

Both cell therapies and RNA-based treatments are advancing toward industrialization, though several bottlenecks must be overcome. Cell therapies encounter obstacles like production complexity, cost, and regulatory hurdles, while RNA technologies struggle with storage stability, scalability, and delivery issues. However, these challenges are integral to the industrialization process. To overcome them, China is focusing on improving manufacturing capabilities, refining regulatory frameworks, and fostering collaborations to accelerate the commercialization of these therapies.

#### 2.2.2 Breakthroughs in vaccines and antibody drug technologies

**FIGURE 3:**
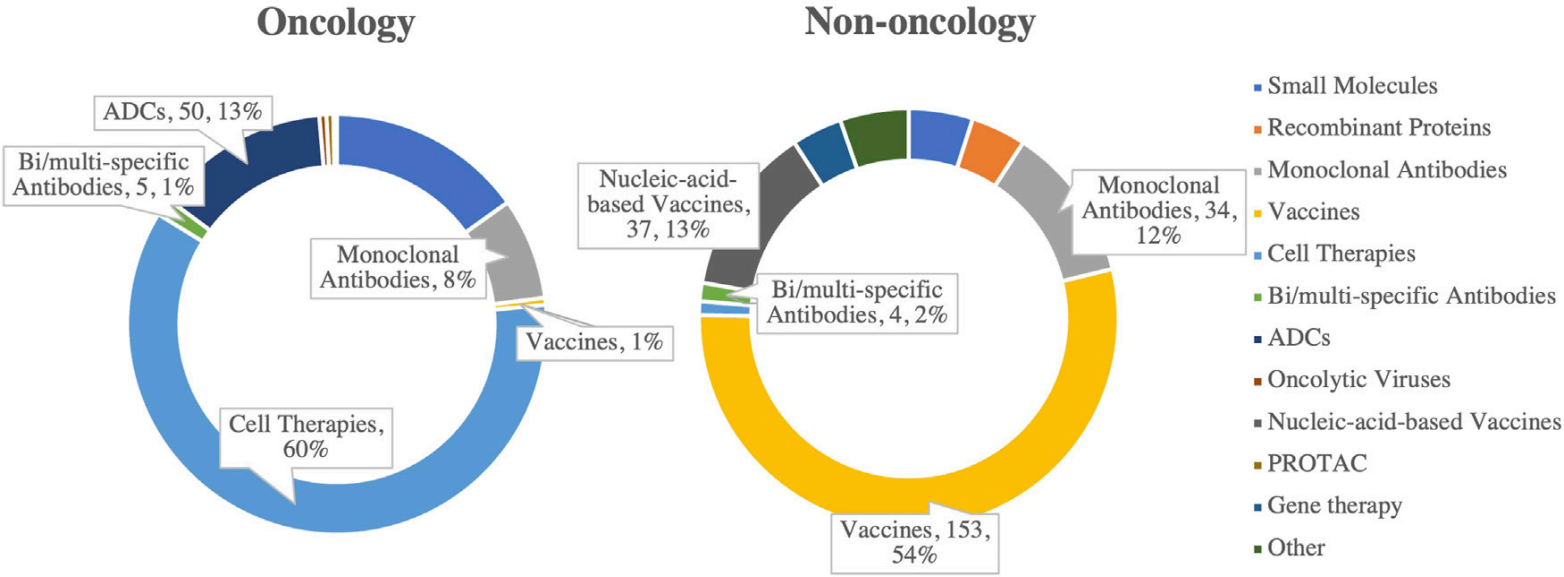
Distribution of drug types among the top 10 R&D pipeline targets in oncology and non-oncology fields in China, 2024. (Source: Analysis based on pharmcube data).

Driven by the pandemic and rising public health demands, the market for vaccines and antibody drugs has experienced significant growth. In China’s drug R&D pipelines, vaccines account for 67% of the top 10 non-oncology targets in 2024, while antibody drugs represent 12% (Figure 3), underscoring their critical role in disease prevention and treatment (Chen R. et al., 2024). These advancements not only address infectious diseases but also provide a foundation for therapies targeting autoimmune diseases, cancer, and chronic conditions.

Technological innovation is the key driver behind this rapid progress. Breakthroughs in new technologies, such as nucleic acid vaccines and bispecific/multispecific antibodies, have not only enhanced efficacy and production efficiency but also expanded their potential applications (Yang and Cui, 2024). For instance, nucleic acid vaccines enable the rapid production of highly specific antibodies targeting disease-causing pathogens, significantly improving the pharmacodynamics of vaccine responses. In oncology, while cell therapies currently dominate (accounting for 60%), antibody drugs are increasingly playing a significant role (Wei et al., 2022). With high technological barriers and considerable efficacy, vaccines and antibody drugs are poised to continue growing in both global and Chinese markets.

#### 2.2.3 Radiopharmaceutical innovations leading the future of precision medicine

Radiopharmaceuticals, with their unique advantages in precise diagnosis and targeted therapy, are emerging as a key focus in pharmaceutical development, driven by the growing trend of integrated diagnosis and treatment. By combining radioactive isotopes with targeting molecules, radiopharmaceuticals enable precision diagnosis and therapy at the molecular level, offering unparalleled value, particularly in the treatment of cancer and neurodegenerative diseases (Huang and Huang, 2024). From a pharmacological standpoint, radiopharmaceuticals enable more specific drug targeting, thereby reducing side effects and improving therapeutic outcomes.

Currently, China’s radiopharmaceutical industry is at a critical stage of rapid growth, characterized by low market penetration but substantial potential. The market size is projected to expand from $840.96 million in $2024 to $1.894 billion by 2031, with a compound annual growth rate (CAGR) of 12.8% (Anushka, 2024). Supported by both policy initiatives and technological advancements, programs like the “Medium and Long-term Development Plan for Medical Isotopes (2021–2035)” are accelerating localization, streamlining approval processes, and optimizing the radiopharmaceutical supply chain. However, transportation and storage of radiopharmaceuticals continue to present significant challenges, due to stringent requirements for radiation shielding, which complicate distribution and accessibility, particularly in remote areas. Furthermore, measures such as decentralizing PET-CT equipment approvals and expanding insurance coverage are unlocking additional market potential, further supporting the availability and accessibility of precision therapies in both domestic and international markets.

#### 2.2.4 Advancements in drug delivery technologies driving high-end formulation innovation

The implementation of policies like consistency evaluation of generic drugs and the National Volume-Based Procurement (NVBP) has compressed profit margins for generic drugs, pushing pharmaceutical companies to seek differentiation as a competitive strategy. High-end formulations have thus emerged as a core focus of transformation. Drug delivery systems, including liposomes, microspheres, and controlled-release formulations, are gaining prominence in drug development due to their ability to enhance bioavailability, improve patient compliance, and meet complex clinical needs (Khadam et al., 2024). These technologies are revolutionizing drug delivery by ensuring that active pharmaceutical ingredients (APIs) are delivered more precisely and effectively to the target areas.

**FIGURE 4:**
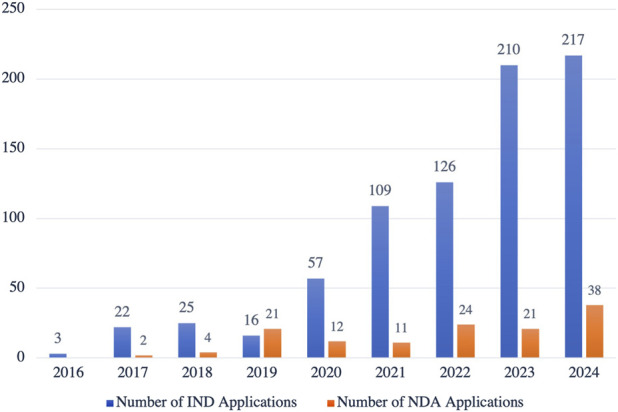
Growth in New Drug Applications for Complex Formulations (Class 2.2) Accepted by the CDE in China, 2016–2024. This figure highlights the increasing number of IND (Investigational New Drug) and NDA (New Drug Application) submissions specifically for complex formulations in China. The steady growth in both IND and NDA submissions reflects the accelerating development of innovative complex formulations, showcasing a clear trend toward advancing this high-value segment of pharmaceutical innovation. (Source: CDE, as of 8 November 2024).

Globally, drug development is shifting from simply addressing basic healthcare needs to improving patient experience and quality of life. High-end formulations have become the focal point of this innovation^(Martin et al., 2024)^. However, in China, the market share of high-end formulations currently stands at less than 3%, far below the international average of 10%, which signals significant growth potential. Supported by initiatives like the 14th Five-Year Plan, the government is encouraging greater investment in complex formulation technology R&D. Since 2016, the number of new drug applications for Class 2.2 complex formulations accepted by China’s Center for Drug Evaluation (CDE) has steadily increased, reflecting both technological advancements and dynamic R&D activity in this sector (Figure 4). In the future, fueled by policy support, evolving market demand, and technological breakthroughs, China’s high-end formulation market is poised for rapid growth and will likely become a major driver of the global expansion of the pharmaceutical industry.

## 3 Trends in the pharmaceutical industry

China’s pharmaceutical industry is undergoing transformative shifts driven by demographic changes, evolving market needs, and technological advancements. The demand for chronic disease treatments and specialty drugs is rising alongside the increasing burden of an aging population. At the same time, pharmaceutical companies are responding to this shift by innovating through biologics, high-end formulations, and data-driven solutions. This section delves into how these trends are reshaping the industry, focusing on the implications for drug access, innovation, and policy-making.

### 3.1 Structural shifts driven by market demand

#### 3.1.1 Aging population driving chronic disease and longevity drugs demand

**FIGURE 5:**
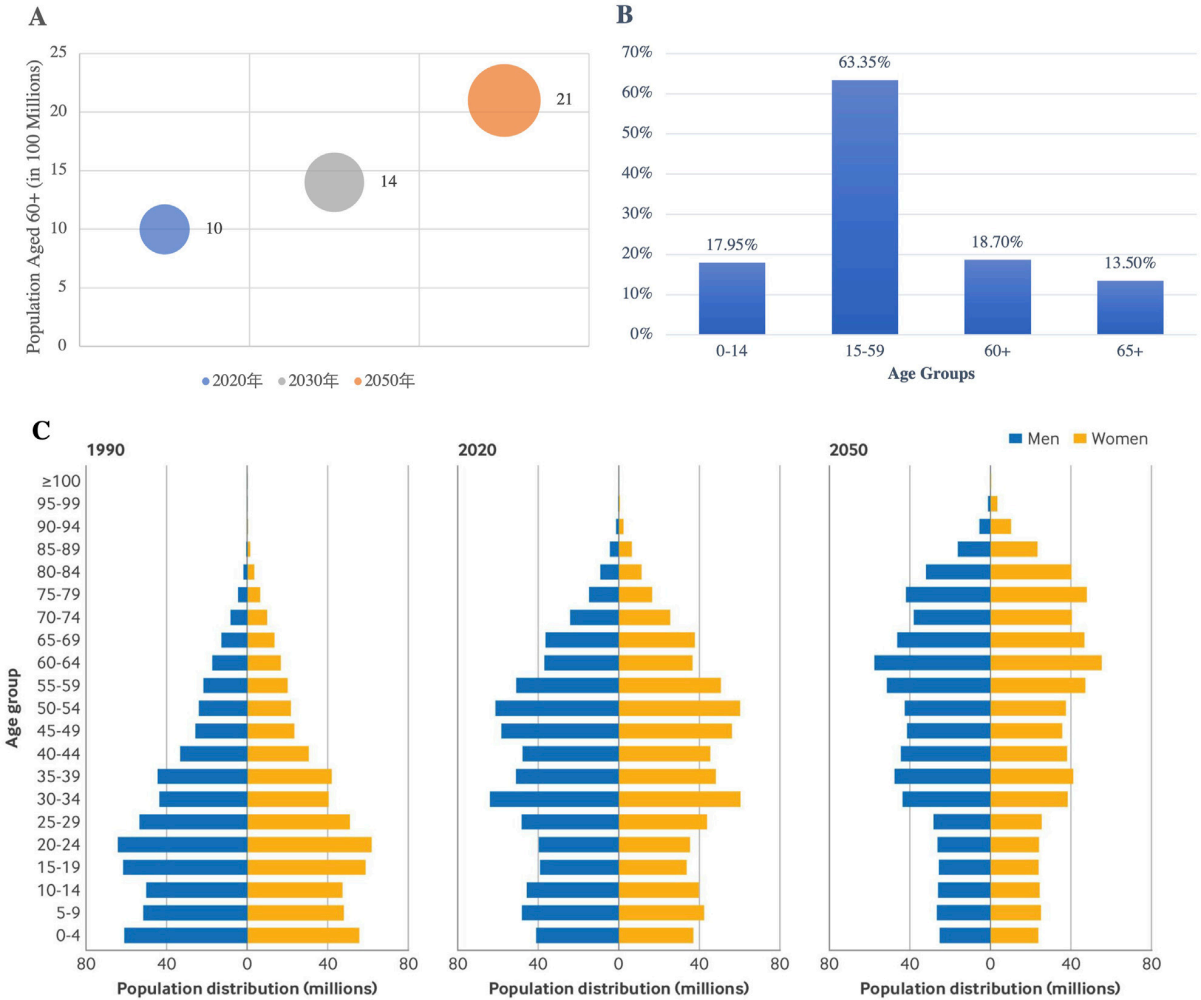
WHO Global Elderly Population Projections and China’s Age Structure Changes (1990–2050): **(A)** The global population aged 60 and over will double by 2050; **(B)** China’s Age Structure According to the Seventh Population Census (2021); **(C)** Population Distribution by Gender and Age in 1990, 2020, and 2050 in China (Lv et al., 2024). (Source: WHO, seventh National Census Report of China, BMJ 2024;387:bmj-2023–076529).

The aging global population is profoundly influencing the pharmaceutical market, driving an ongoing increase in the demand for chronic disease management and longevity treatments. According to the World Health Organization (WHO), the global population aged 60 years and older is projected to rise from one billion in 2020 to 1.4 billion by 2030, and further to 2.1 billion by 2050, with the number of individuals aged 80 years and older expected to reach 426 million by 2050 (WHO, 2024). In China, this aging trend is even more pronounced, with 15.4% of the population aged 65 years and older in 2024, and an estimated elderly population expected to exceed 400 million by 2035 (Figure 5) (Lv et al., 2024). Furthermore, health data from China Health Management (CMH) shows a significant rise in morbidity rates, with the 2-week morbidity rate among both urban and rural residents predicted to reach 44.2% by 2033, reflecting the growing burden of chronic diseases across diverse populations (CMH, 2021; Su et al., 2023).

This demographic shift is contributing to a rise in cardiovascular diseases, metabolic disorders, and neurodegenerative conditions, which are placing a significant burden on healthcare systems. The growing prevalence of non-communicable diseases such as hypertension and diabetes is further driving the demand for related treatments (Lv et al., 2024). To meet these evolving needs, pharmacological innovations are essential in advancing drug development. Moreover, policy interventions will play a critical role in ensuring equitable access to these treatments. National health policies must strike a balance between fostering innovation and ensuring the affordability and accessibility of advanced therapies, including monoclonal antibodies and cell therapies.

Pharmaceutical companies are responding to these challenges by intensifying R&D efforts in chronic disease management, as well as making breakthroughs in technologies like monoclonal antibodies and cell therapies. Health management platforms that leverage AI and big data are improving the precision of disease prevention and chronic disease management strategies. Meanwhile, longevity drugs and health supplements aimed at delaying aging and enhancing immune function have emerged as key areas of R&D, driven by advancements in technologies such as telomerase activators, NAD + metabolism regulators, and anti-inflammatory agents. Looking ahead, the integration of chronic disease management, longevity therapies, and preventive medicines presents enormous potential not only to meet the needs of aging populations but also to offer innovative pharmaceutical companies a competitive edge in the future market. Nevertheless, overcoming the challenges of market access, cost containment, and regulatory hurdles will be critical in realizing this potential at scale.

#### 3.1.2 Decelerating growth of oncology drug pipelines and diversification of R&D priorities

**FIGURE 6:**
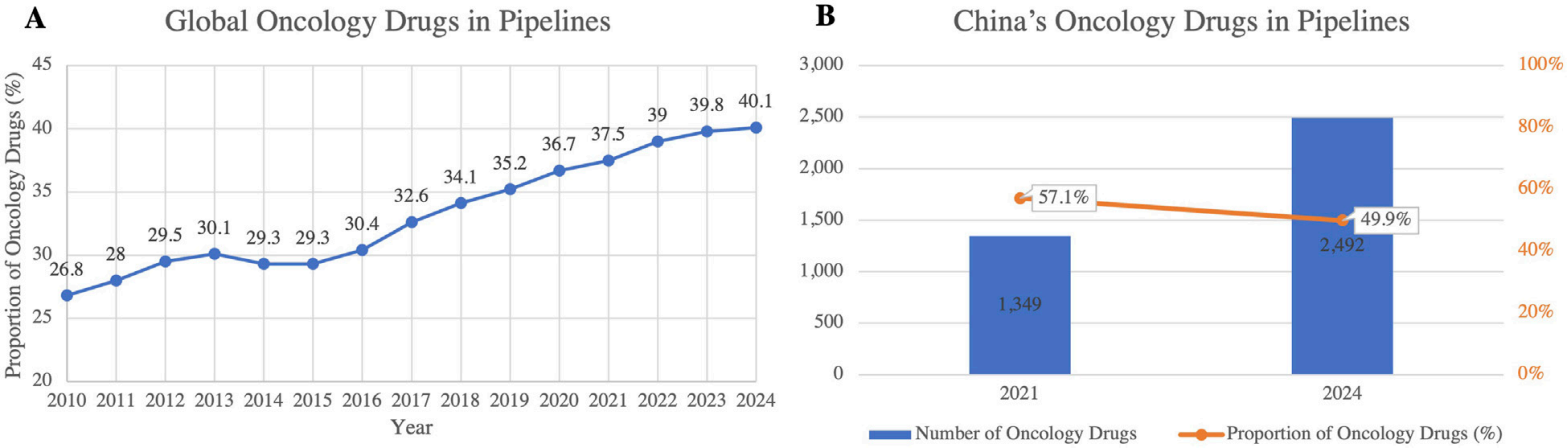
**(A)** Trends in Global Oncology Drug Pipeline Share (2010–2024); **(B)** Number and Proportion of Oncology Drugs in China’s R&D Pipelines (2021 and 2024). This figure highlights the consistent growth of oncology drugs as a proportion of the global drug pipeline, reaching 40.1% in 2024. In China, the number of oncology drugs in the pipelines nearly doubled between 2021 and 2024, but their proportion declined from 57.1% to 49.9%, indicating a shift toward more diversified drug development. (Source: Pharmaprojects, 2024)

Global anticancer drug research continues to advance, but its growth trajectory is slowing. Although oncology still occupies a dominant position in R&D pipelines, its expansion is approaching maturity, reflecting wider patient access to treatments (Figure 6A) (ITELINE, 2025). In China, oncology pipelines have grown sharply, from 1,349 projects in 2021 to 2,492 in 2024, an increase of 84.7% over the period. However, their share of the total pipeline declined by 7.2 percentage points (Figure 6B), indicating a gradual focus shift in industry priorities toward diversification (Kong et al., 2023; Chen Z. et al., 2024). The concentration on mature targets has also intensified competition and product homogenization, diminishing the prospects for breakthrough innovation.

As competition in the oncology sector intensifies, R&D is increasingly directed towards cardiovascular, respiratory, and metabolic diseases (Sheng-Shou, 2023). This transition reflects global health priorities and the need to address the rising burden of chronic and lifestyle-related diseases. For China, this pivot opens new opportunities to align with emerging health demands and to broaden its innovation landscape. At the same time, the trend reduces the risks associated with over-concentration in oncology and encourages a more balanced allocation of industry resources.

From a policy perspective, this diversification requires stronger support mechanisms. While oncology will remain a key therapeutic area, underdeveloped fields such as chronic and metabolic disorders, which are now becoming leading causes of morbidity and mortality globally, demand greater incentives through public–private partnerships, targeted subsidies, and tax benefits. Such measures will help overcome funding gaps and accelerate innovation beyond oncology. For Chinese pharmaceutical companies, this strategic shift enhances their competitiveness, both domestically and globally, by positioning them as contributors to underexplored yet high-burden therapeutic areas.

Nevertheless, challenges remain. Oncology continues to attract the bulk of investment, which may limit the pace of diversification. Moreover, R&D in cardiovascular and metabolic diseases often requires long clinical cycles, substantial patient populations, and complex trial designs, which may slow the return on investment. Balancing the pursuit of oncology innovation with the expansion into other therapeutic areas will therefore be critical for sustaining growth and achieving broader public health benefits.

#### 3.1.3 Rising demand for rare diseases and specialty drugs

Driven by evolving medical needs and technological advancements, the markets for rare disease and specialty drugs are experiencing rapid growth. Although patients with rare diseases represent a small portion of the global population, their absolute number in China is substantial, and the unmet medical needs remain vast. From a pharmacological perspective, rare diseases are often complex and heterogeneous, posing unique challenges for drug discovery and development. Despite these challenges, advancements in genomic medicine, gene therapy, and biologics are opening new avenues for treatment.

**FIGURE 7:**
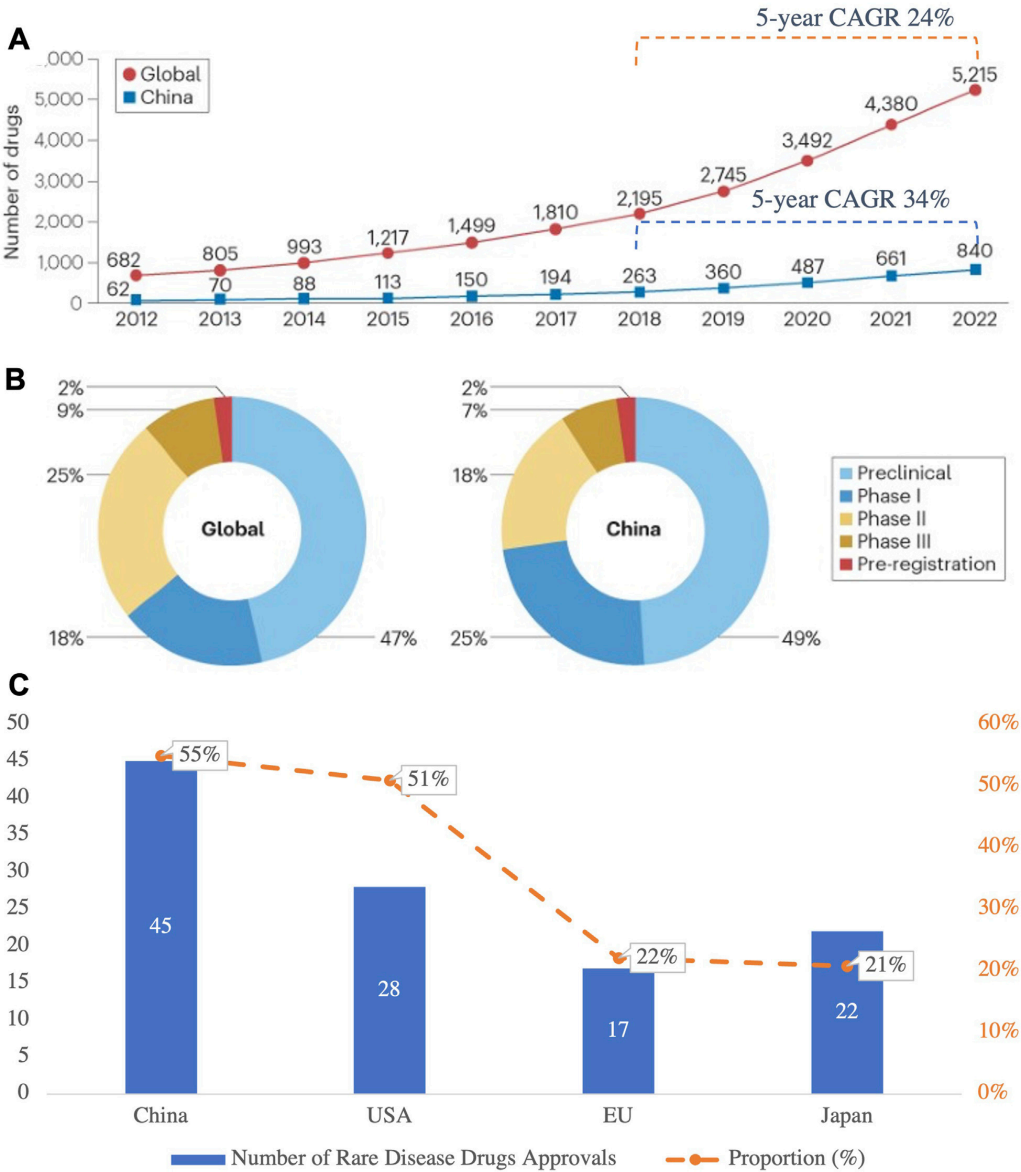
**(A)** Growth of Rare Disease Drug Pipelines (2012–2022), **(B)** R&D Stage Distribution of Rare Disease Drugs in China and Globally; **(C)** Number of Rare Disease Drugs Approved and Their Proportion in Total Drug Approvals in 2023 in Major Countries and Regions. This figure highlights the rapid growth of rare disease drug pipelines in China and globally, with China’s pipelines achieving a 5-year CAGR of 34%, outpacing the global CAGR of 24%. While the R&D stage distribution is similar between China and the global landscape, the proportion of preclinical and clinical-stage drugs in China remains slightly higher. In 2023, China led in the number of rare disease drug approvals, contributing 55% of all approvals, showcasing its expanding role in addressing rare disease needs. (Source: Nature Reviews Drug Discovery 2024; NMPA).

Globally, investment in R&D for rare disease drugs continues to increase, reflecting greater attention to these unmet medical needs. In China, from 2018 to 2022, the 5-year CAGR of rare disease drug pipelines reached 34%, notably exceeding the global average of 24% (Figure 7A) (Chen R. et al., 2024). By the end of 2022, China had 840 rare disease drugs in development, with clinical-stage distribution aligning closely with global trends (Figure 7B) (Chen Z. et al., 2024). This surge in R&D activities reflects the pharmacological innovations propelling the treatment of rare diseases and highlights China’s growing role in the global pharmaceutical landscape.

In 2023, 45 rare disease drugs were approved for marketing in China, accounting for 55% of all new drug approvals that year, a remarkable achievement driven by robust policy support for rare disease R&D (Figure 7C) (Shaohong et al., 2024). Regulatory changes, such as expedited approval pathways for orphan drugs and innovative pricing models, have significantly contributed to these advancements. These policy shifts are not only accelerating drug development but also enhancing the affordability and accessibility of treatments, aligning with broader efforts to improve healthcare outcomes.

However, challenges persist, especially in pediatric medications, a critical category of specialty drugs. Due to specific requirements and high technical barriers, pediatric drugs face significant supply shortages (Chen P. et al., 2021). Developing pediatric medications presents the dual challenge of ensuring safety and efficacy for younger populations, often necessitating specialized formulations and dosages. With enhanced policy support and increased R&D investment, companies are intensifying efforts in pediatric drug development, which will improve the overall medication supply system, ensuring more effective treatments for children with complex health conditions.

The growing demand for rare disease and pediatric drugs is unlocking new market opportunities and driving a shift toward more technology-intensive and higher-value sectors (Zhao et al., 2023). Pharmaceutical companies are capitalizing on cutting-edge technologies like CRISPR gene editing and advanced biologics to develop targeted therapies that address the root causes of diseases, rather than merely managing symptoms. With strong policy backing and ongoing technological innovation, this sector is well-positioned to become a key growth area for China’s pharmaceutical industry in the future.

### 3.2 Pharmaceutical industry upgrading and technological innovation-driven growth

#### 3.2.1 Biologics, TCM, and innovative drugs driving industry transformation

Although chemical drugs continue to dominate the pharmaceutical market, their growth prospects have diminished due to shrinking profit margins and increased competition (Zheng et al., 2023). This shift highlights the growing importance of innovative drug development, particularly in biologics and traditional Chinese medicine (TCM). In the future, differentiated competition and innovative R&D will be essential strategies for chemical pharmaceutical companies.

**FIGURE 8:**
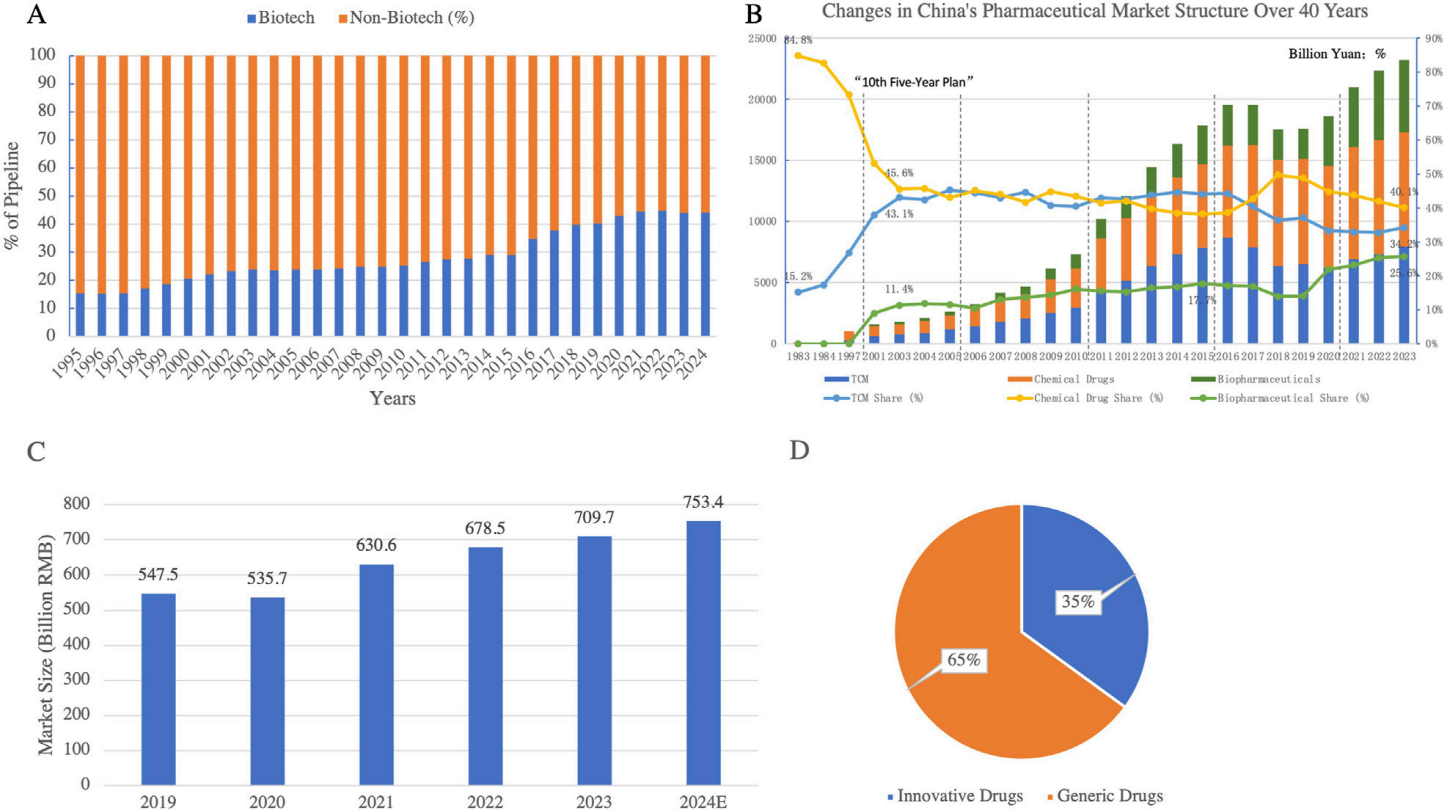
**(A)** Biological vs non-biological drugs as a percentage of the pipeline Globally (1995–2024); **(B)** Evolution of China’s Pharmaceutical Market Structure Over 40 Years of Reform and Opening-Up; **(C)** Market Size of Innovative Drugs in China (2019–2024); **(D)** Market Structure of China’s Pharmaceutical Industry in 2023. This figure shows the steady rise of biological drugs as a percentage of the global drug pipeline over the past 3 decades. Biotech drugs have grown significantly, reaching approximately 40% of the pipeline by 2024, reflecting the increasing focus on innovative biologics in drug development. The proportion of biological drugs in the market structure in China is also gradually rising. Figures C and D highlight the steady growth of China’s innovative drug market, projected to reach 753.4 billion RMB by 2024. In 2023, innovative drugs accounted for 35% of the total pharmaceutical market, reflecting a gradual shift toward high-value innovative therapies while generic drugs still dominate the market at 65%. (Data Source: *Pharmaprojects, 2024;* Annual Chinese Pharmaceutical Yearbook; China Pharmaceutical Enterprises Association; Frost & Sullivan).

Biologics are rapidly gaining prominence, fueled by cutting-edge technologies such as gene therapy and cell therapy. Their share in global drug pipelines is approaching 45% and continues to grow (Figure 8A) (Martins et al., 2022). Biologic drugs, including monoclonal antibodies, gene therapies, and therapeutic proteins, are revolutionizing treatment strategies for various conditions, especially cancers, autoimmune diseases, and genetic disorders. In China, the proportion of biologics applications has been steadily increasing, indicating significant potential in this sector. Notably, breakthroughs in gene editing technologies like CRISPR have accelerated the development of biologics, enabling faster and more targeted treatments.

TCM, although its market share has relatively declined, is gaining renewed attention as health management needs grow in the post-pandemic era. The integration of TCM with modern pharmacology is driving innovations in personalized medicine. Through the study of bioactive compounds in herbal medicine, TCM is increasingly being recognized for its potential in preventing and managing chronic diseases, with its bioactivity being scientifically validated. Policy support, including favorable regulations and funding for research, is guiding TCM towards high-quality development, pushing it to adapt to modern healthcare systems while retaining its traditional roots.

The innovative drug market has seen remarkable growth. Data shows that China’s innovative drug market expanded from 550.6 billion RMB in 2019 to 711.3 billion RMB in 2023, and is projected to reach 753.4 billion RMB by 2024, capturing 35% of the market share (Figures 8C,D) (Institute, 2024). This growth is being fueled by the introduction of biologics, personalized medicine, and targeted therapies, which have higher efficacy profiles and are gaining increasing global acceptance. With the support of policy and technology, the market share of innovative drugs is expected to surpass 50%, becoming the core driver of pharmaceutical industry transformation.

Overall, the growing prominence of biologics and TCM is gradually diminishing the dominance of traditional chemical drugs (Figure 8B). This reflects the shift from small-molecule chemical drugs to more advanced biologic therapies. Looking ahead, these three sectors-chemical drugs, TCM and biologics-are expected to form a tripartite market structure, driving the Chinese pharmaceutical industry towards diversification and high-value growth, while enhancing global competitiveness.

#### 3.2.2 Rising mergers and acquisitions catalyzing pharmaceutical industry consolidation

**FIGURE 9:**
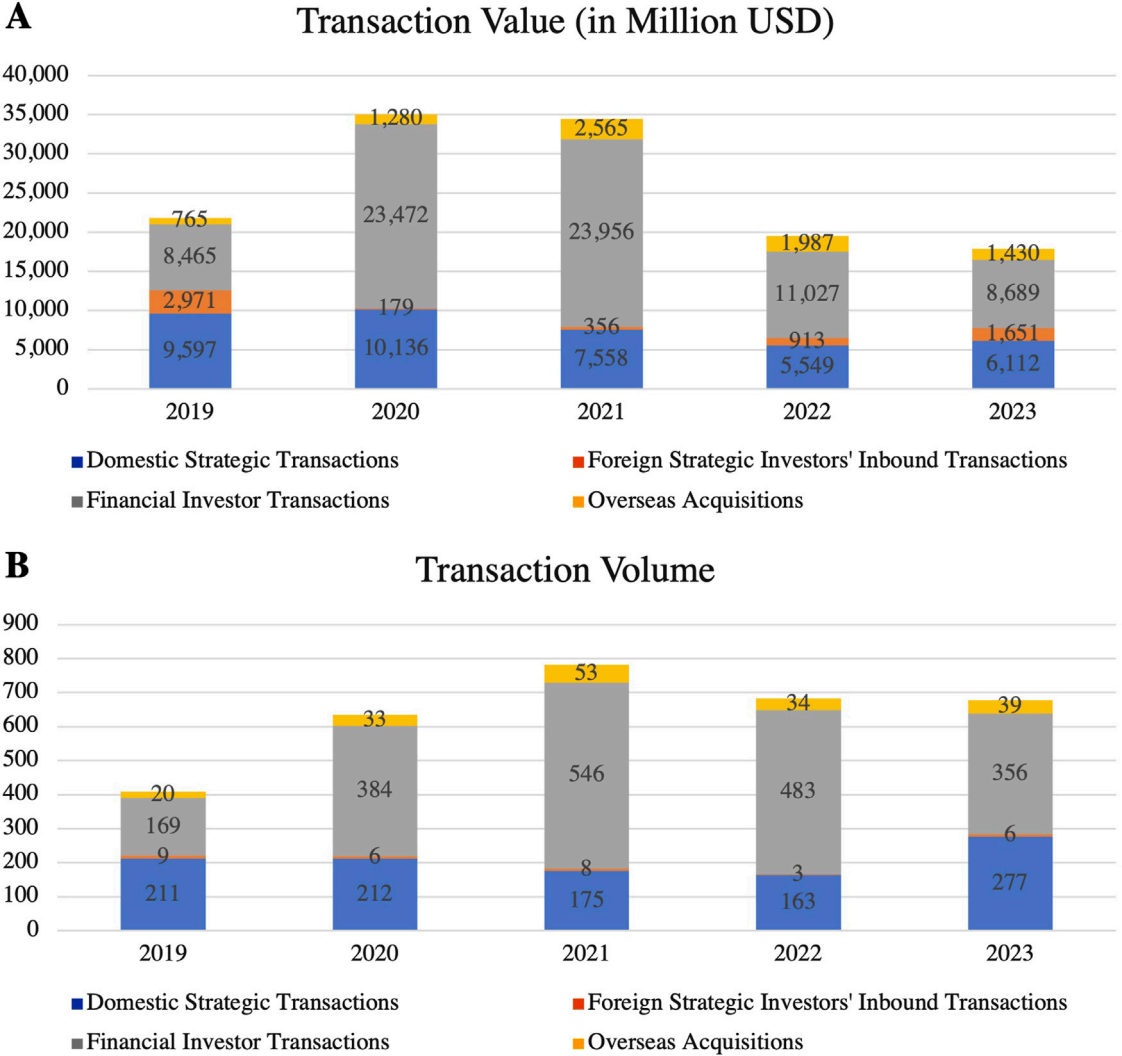
M&A **(A)** Deal Value and **(B)** Volume in China’s Pharmaceutical Industry (2019–2023). These figures highlight the trends in M&A in China’s pharmaceutical industry. While the transaction value peaked in 2021, both value and volume have shown a decline in 2022 and 2023, indicating a cooling M&A environment. (Source: Thomson Reuters, PitchBook, PwC Analysis).

Driven by technological advancements and intensified market competition, mergers and acquisitions (M&A) in China’s pharmaceutical industry are accelerating. In 2023, the number of domestic strategic investments rose by 70% year-on-year, while the volume and value of foreign inbound investments also saw significant increases (Figure 9) (PwC, 2024).

In September 2024, the China Securities Regulatory Commission introduced reforms to the M&A market, further stimulating the capital market and accelerating the growth in M&A activity. These regulatory reforms aim to create a more favorable environment for pharmaceutical companies to secure financing and resources for innovation. The surge in M&A activity reflects both capital operations and fierce competition for technological and market resources. In 2024, multinational pharmaceutical companies reached 71 acquisition agreements with Chinese entities, a 37% increase from the 52 agreements recorded 4 years earlier. This reflects a notable acceleration in M&A activity as multinational companies seek to address patent expirations and secure new growth opportunities by acquiring local Chinese firms (Harachand, 2024). By doing so, they inject both capital and advanced technologies into the rapidly growing Chinese pharmaceutical market.

Meanwhile, Chinese domestic companies are expanding their business operations and upgrading industrial capabilities through M&A. By acquiring smaller firms or forming strategic alliances, domestic companies are enhancing their resilience and strengthening their R&D capabilities. Small and medium-sized enterprises (SMEs) are leveraging partnerships to ease resource constraints and enhance their ability to innovate. Moving forward, M&A-driven economies of scale and resource optimization will lead to greater market consolidation and competitiveness in the pharmaceutical sector. However, the frequent acquisitions by multinational companies may challenge the independent innovation of local firms, posing a critical issue for the industry to balance capital influx with innovation development.

#### 3.2.3 Emerging trends in marketing and business model innovation

**FIGURE 10:**
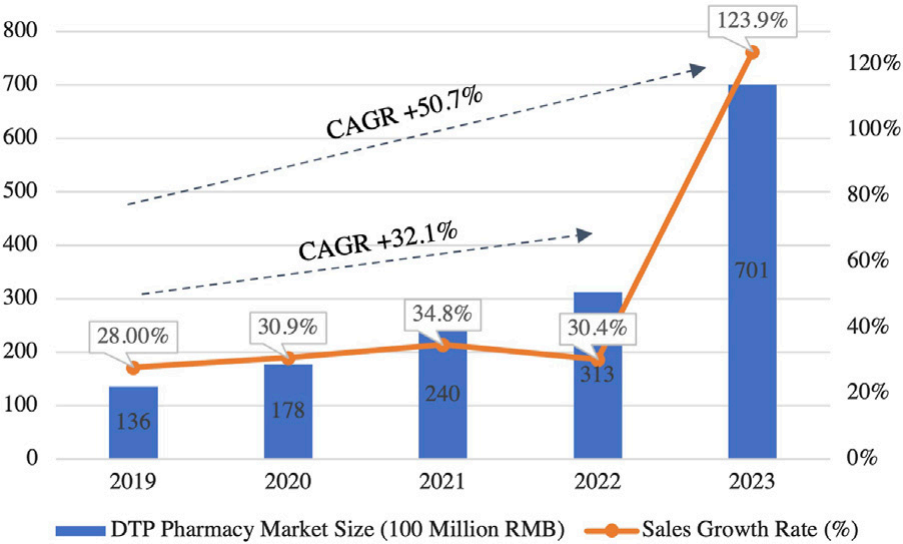
Market Scale and Growth Trends of China’s DTP Pharmacy (2019–2023). This figure illustrates the rapid expansion of China’s DTP pharmacy market. The market size has grown significantly, with a CAGR of 50.7% between 2019 and 2023, reflecting the increasing demand for specialty drugs and outflow prescriptions in the retail pharmacy sector. (Source: Pharbers Data).

Policies such as the NVBP have significantly reshaped traditional pharmaceutical marketing practices, pushing the industry toward a new era of prescription outsourcing. In parallel, the ‘Internet+' initiative has accelerated the pharmaceutical sector’s digital transformation, fostering the rise of innovative business models that integrate online and offline channels, reshaping the operational framework of the industry. A key example of this transformation is the rapid growth of Direct-to-Patient (DTP) pharmacies. The DTP market expanded from 13.6 billion RMB in 2019 to 70.1 billion RMB in 2023, reflecting a 5-year CAGR of 50.7% (Figure 10) (Chen Z. et al., 2021). DTP pharmacies play a pivotal role in improving patient access to specialty drugs and efficiently managing outflow prescriptions, becoming a cornerstone of China’s pharmaceutical digital transformation, (Manzhu et al., 2022).

Meanwhile, offline retail pharmacies are adapting to the changing landscape by expanding their services to include outpatient care and embracing O2O (Online-to-Offline) business models. This transition enhances drug purchasing convenience and optimizes the patient experience, reflecting the pharmaceutical industry’s move towards more integrated, patient-centered service offerings (Simeng and Shuling, 2024). Looking forward, the ‘Internet+' model will continue to evolve, with omnichannel strategies and data-driven precision marketing emerging as core components of pharmaceutical marketing. These trends highlight the increasing importance of technology and data analytics in personalizing patient care and optimizing drug delivery. This shift will enable companies to better meet diverse patient needs, accelerate the modernization of pharmaceutical marketing, and inject new momentum into China’s pharmaceutical sector.

### 3.3 Global expansion and international competition in the pharmaceutical industry

#### 3.3.1 Growing international competition and China’s expanding independent R&D

Global competition in the bioeconomy is intensifying, with developed nations such as the U.S. and the EU strengthening their policy frameworks to solidify their dominance. For instance, in 2024, the U.S. enacted the *Biotechnology Safety Act*, aimed at ensuring the safety and sustainability of biotechnology innovations. This regulatory shift has further escalated global competition, particularly in biopharmaceuticals, as countries race to secure leadership in emerging medical technologies.

**FIGURE 11:**
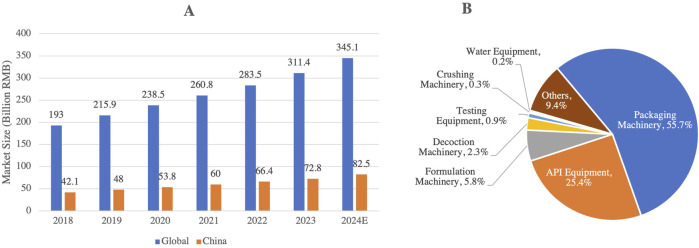
**(A)** Growth of the Pharmaceutical Equipment Market in China (2018–2024), and **(B)** Composition and Market Share of Pharmaceutical Manufacturing Equipment in China (2023). This figure highlights the growth of China’s pharmaceutical equipment market, which is projected to reach 82.5 billion RMB by 2024. The pie chart B reveals that the market is primarily concentrated in packaging machinery (55.7%) and API equipment (25.4%), while testing and high-end pharmaceutical equipment hold a relatively low share, indicating potential areas for technological advancement and market development (Source: www.askci.com).

In response to these challenges, China’s pharmaceutical industry is accelerating its independent R&D capabilities, with a notable shift from generic to innovative drug development. The number of innovative drugs launched in China has risen dramatically, from 11 in 2019 to 46 in 2024. This surge is largely driven by the country’s growing capabilities in biologics, targeted therapies, and regenerative medicine, alongside substantial investments aimed at enhancing China’s biotechnology infrastructure. Moreover, the pharmaceutical equipment market in China has experienced notable growth, expanding from 30.7 billion RMB in 2016 to 72.8 billion RMB in 2023 (Figure 11A), reflecting the results of industrial upgrades and the increasing sophistication of local pharmaceutical manufacturing.

Despite these advances, however, China still faces challenges regarding its dependence on imports for high-end pharmaceutical equipment and diagnostic instruments, which continued to account for a substantial portion of the market share in 2023. Notably, drug packaging machinery and raw material pharmaceutical machinery represented 55.7% and 25.4% of China’s pharmaceutical equipment market, respectively (Figure 11B), underscoring the ongoing need for core technology localization. The 14th Five-Year Plan places a strong emphasis on policy support for drug innovation and the local development of pharmaceutical equipment to reduce reliance on foreign imports and enhance technological autonomy (Xu et al., 2021). Looking ahead, ongoing technological breakthroughs and industrial restructuring are expected to further bolster China’s global competitiveness in the biopharmaceutical industry, positioning the country as a key player in the international market.

#### 3.3.2 Expanding international cooperation and advancing multicenter clinical trials

**FIGURE 12:**
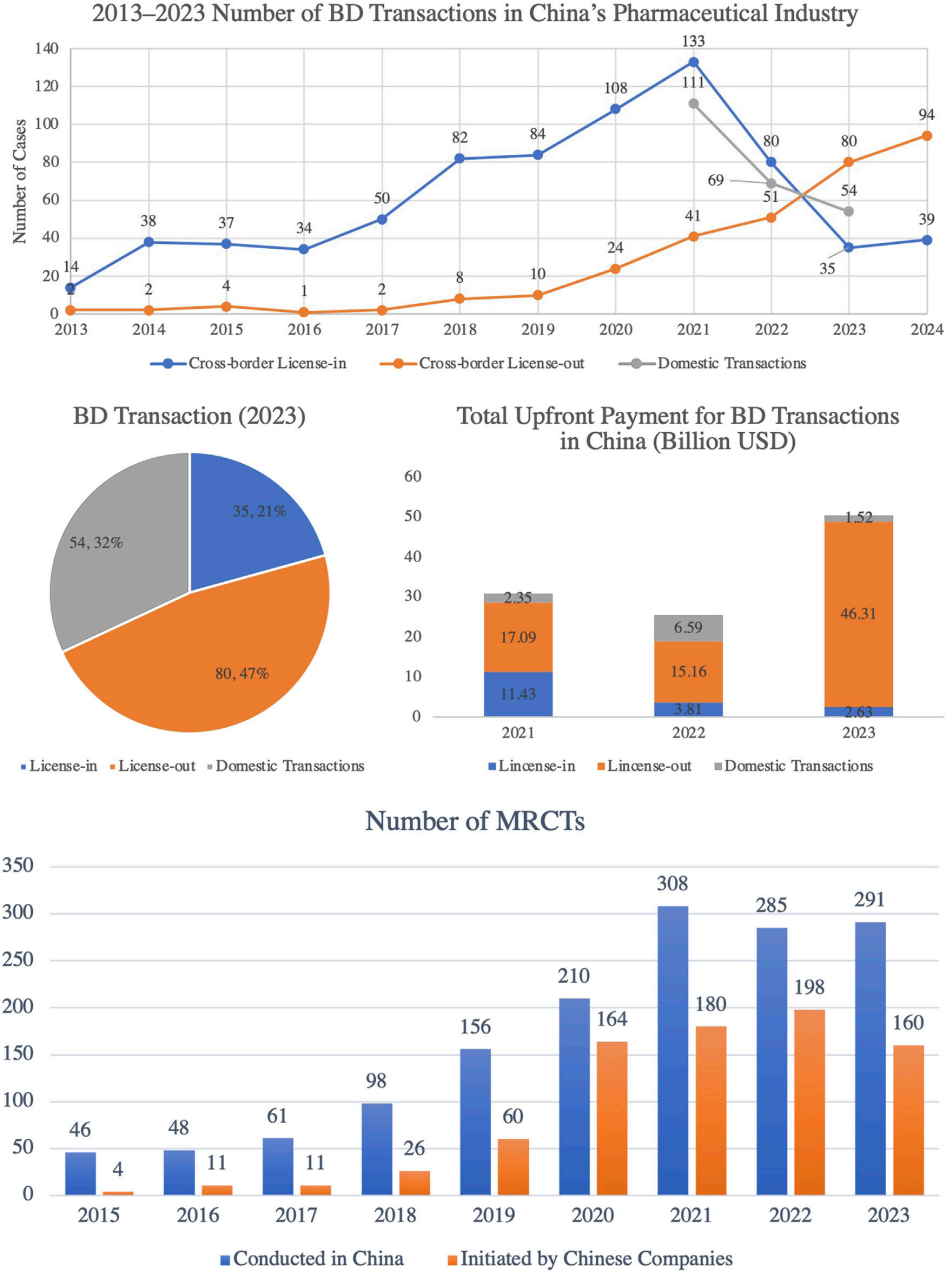
Trends in Pharmaceutical Business Development (BD) Transactions and Multi-Center International Clinical Trials (MRCTs) in China, 2015–2023. This figure illustrates the rapid growth in both pharmaceutical BD transactions and MRCTs in China. The pharmaceutical BD transactions in China have experienced significant growth, particularly in cross-border license-out deals, which have risen steadily since 2017. By 2023, license-out transactions accounted for 47% of all deals, marking a significant increase in the upfront payments for these transactions, underscoring the growing global recognition of Chinese pharmaceutical assets. Simultaneously, the number of MRCTs conducted in China has steadily increased, reaching 291 in 2023, highlighting China’s growing significance as a global clinical trial hub. Furthermore, MRCTs initiated by Chinese companies have also expanded, reaching 160 trials in 2023, which reflects the rising international presence of Chinese pharmaceutical companies. (Source: TrialCube™ Database, Frost & Sullivan).

Chinese pharmaceutical companies are accelerating their international expansion through a dual strategy of ‘going out’ and ‘bringing in’ cooperation. In 2023, cross-border license-out transactions exceeded license-in transactions for the first time (Figure 12), covering cutting-edge fields such as antibody drugs and cell therapies (MyBioGate, 2024). This shift underscores China’s growing global influence in biotechnology, as the country increasingly plays a central role in licensing innovative treatments for international markets.

In 2024, license-out transactions continued to thrive, with 94 deals finalized, reflecting significant progress in securing R&D funding and technological partnerships with multinational companies. Meanwhile, China’s participation in international multicenter clinical trials (MRCTs) has grown rapidly, with the number of trials it leads increasing from four in 2015 to 160 in 2023 (Figure 12). This growth reflects China’s expanding role in advancing the internationalization of innovative drugs. The involvement in MRCTs accelerates the development of global treatment standards, enhances the credibility of Chinese clinical data, and facilitates the entry of Chinese innovations into global markets.

Furthermore, multinational pharmaceutical companies are increasingly investing in and acquiring Chinese companies to access innovative technologies. For instance, BioNTech acquired Pumis Biotech for $800 million in 2023, underscoring the global appeal of Chinese biotech technologies. These acquisitions reflect a strategic integration of local innovations with global pharmaceutical resources, allowing multinational companies to tap into China’s burgeoning biotech sector and strengthen their competitive positions (Cheung, 2024).

Looking ahead, external licensing agreements will enable more Chinese innovative drugs to enter international markets. Furthermore, the expansion of MRCTs will further solidify China’s presence in global clinical trials. The growing trend of multinational M&A will continue to integrate local technologies with international expertise, accelerating the global recognition of China’s pharmaceutical innovations and securing greater influence in the worldwide industry.

#### 3.3.3 China’s pharmaceuticals industry adopting EV industry strategies for global expansion

Drawing inspiration from the success of the electric vehicle (EV) industry, the Chinese pharmaceutical sector is increasingly leveraging technological innovation and strategic global expansion to penetrate international markets. Much like the EV industry, which capitalized on advancements in clean energy technology to break into international markets, China’s pharmaceutical industry is now harnessing cutting-edge biopharmaceutical technologies to position itself on the world stage.

On the one hand, innovative drugs are capitalizing on technological and cost advantages to enter high-value markets such as Europe and the U.S., where regulatory hurdles and fierce competition are particularly challenging. On the other hand, China’s pharmaceutical sector is also expanding rapidly into emerging markets. The Belt and Road Initiative (BRI) has played a crucial role in facilitating Chinese companies’ expansion into Southeast Asia and Africa, regions that are becoming increasingly important in global pharmaceutical trade. Between 2015 and 2023, China invested over $6.6 billion in Southeast Asia’s healthcare market, further strengthening the position of Chinese companies within these emerging pharmaceutical supply chains (Xiaohui, 2024). This investment, combined with the strategic advantages of the BRI, has enabled Chinese companies to rapidly integrate into the healthcare ecosystems of these regions.

In the future, the internationalization of China’s pharmaceutical industry is expected to unfold through several key strategies: (1) deepening the penetration of innovative drugs into European and American markets via license-out transactions and participation in international MRCTs; (2) consolidating advantages in emerging economies like Southeast Asia and Africa, where the BRI continues to open new avenues for collaboration and growth; (3) optimizing supply chain efficiency and enhancing international brand recognition to enable differentiated competition, positioning Chinese companies as reliable and competitive players in the global pharmaceutical market.

Despite the significant momentum behind China’s pharmaceutical internationalization, challenges such as regulatory barriers and insufficient brand recognition remain. However, according to McKinsey’s forecast, China’s biopharmaceutical industry is expected to secure a significant global position by 2028 (Company, 2022). Future development will depend on the ability to adapt to international regulatory standards, build a global ecosystem of partnerships, and deepen international cooperation (Company, 2022). By optimizing its strategic layout and enhancing global competitiveness, China’s pharmaceutical industry is poised to replicate the success of the EV industry. As the sector continues to innovate and expand, it is set to become a key player in the global pharmaceutical market, contributing Chinese innovation and expertise to the advancement of global healthcare.

## 4 Discussion

Current policies such as “drug price negotiations” and NVBP have effectively reduced drug prices and alleviated pressure on medical insurance funds. However, these policies have also significantly squeezed corporate profits, leaving many pharmaceutical companies struggling to maintain normal operations and continue investing in R&D. In the worst cases, this could even lead to stagnation in industry development (Zheng et al., 2023). The constraints of the domestic market have prompted many pharmaceutical companies to pursue global expansion in search of new growth opportunities. However, the internationalization of the pharmaceutical sector presents its own set of challenges, including regulatory barriers, market entry restrictions, and financial pressures.

To mitigate these pressures, many companies have licensed out their R&D achievements to foreign entities, resulting in steady increases in such transactions in recent years (MyBioGate, 2024). While this licensing model may generate short-term revenue, it often comes at the expense of potential blockbuster drugs and core technologies, which undermine domestic innovation capabilities. As this issue becomes more apparent, an increasing number of companies are establishing international multicenter clinical trials (MRCTs) to explore independent paths for global expansion. This shift is driving companies to adopt more diversified cooperation models, such as Newco-a business structure involving joint ventures to share risks and resources. This trend is not only an inevitable evolution for the industry but also an urgent issue that requires robust policy support to help Chinese pharmaceutical companies secure a stronger foothold in international markets.

In parallel, the rise of AI-assisted drug development holds significant promise for enhancing R&D efficiency and reducing costs. However, its application is not without challenges, including issues with algorithm reliability, data quality, and unclear industrialization pathways. Moving forward, the rational utilization of AI technology-coupled with traditional R&D practices-will be crucial in driving sustainable industry development. Exploring ways to effectively incorporate AI into the existing pharmaceutical R&D ecosystem will be a critical area of focus.

Despite these advancements, China’s healthcare expenditure as a percentage of GDP remains below the world average, indicating considerable potential for growth in the sector (Data, 2022). However, solely relying on healthcare cost control and drug price reductions is not a sustainable long-term solution. The government should consider a moderate increase in healthcare spending, particularly to support the development of the biopharmaceutical industry. This would allow the country to shift from a cost-driven growth model to one that is innovation-driven. Simultaneously, improving the commercial insurance system is essential for easing the burden on healthcare funds. A diversified payment system should be established to prevent the emergence of low-price, low-quality competition, which often leads to detrimental cycles of price-cutting and quality erosion in the industry. Moreover, more explicit policy guidance is also needed to address homogeneous competition and to encourage the development of high-quality drugs, ultimately bridging the supply gap for quality medications and preventing inferior products from flooding the market. This will ensure a more sustainable and efficient industry structure.

In the future, China’s pharmaceutical industry must strike a delicate balance between healthcare cost control and industrial innovation. Achieving this balance will require optimal resource allocation, with effective coordination between policy and market forces. The government should continue to support R&D for high-quality innovative drugs and facilitate companies’ efforts in their global expansion. By developing a growth framework that prioritizes innovation-driven development and places equal emphasis on both domestic and international dual circulation, China can build a strong and globally competitive pharmaceutical sector.

## 5 Conclusion and perspective

China’s pharmaceutical industry is undergoing a pivotal transformation, driven by advanced technologies such as precision medicine, synthetic biology, and artificial intelligence. These innovations have significantly enhanced the efficiency and precision of drug development, offering new solutions to meet the growing medical needs stemming from an aging population, rising chronic diseases, and the increasing prevalence of rare diseases. Additionally, strong policy support and rising market demand are guiding the industry toward more high-end and differentiated drug categories, marking a shift to value-driven innovation in the industry.

However, despite these promising advancements, significant challenges persist. These include limited innovation capacity, low confidence in capital markets, and a relatively low level of corporate internationalization. To successfully transition from a “generic-driven” to an “innovation-driven” strategy, it is crucial for the government to continue leveraging policy incentives, streamline regulatory processes, and boost R&D funding support. Concurrently, pharmaceutical companies must accelerate the adoption of cutting-edge technologies and expand their global footprint to strengthen their competitiveness in both domestic and international markets.

Looking forward, China’s pharmaceutical industry is poised to enter a new stage of high-quality development, underpinned by technological innovation, the optimization of industrial structures, and internationalization. As the industry progresses, it will contribute not only to global healthcare solutions but also offer Chinese wisdom and innovative approaches to the challenges faced by the global pharmaceutical market.
